# High-Carbohydrate Diet Enhanced the Anticontractile Effect of Perivascular Adipose Tissue Through Activation of Renin-Angiotensin System

**DOI:** 10.3389/fphys.2020.628101

**Published:** 2021-01-15

**Authors:** Daniela Esteves Ferreira dos Reis Costa, Ana Letícia Malheiros Silveira, Gianne Paul Campos, Natália Ribeiro Cabacinha Nóbrega, Natália Ferreira de Araújo, Luciano de Figueiredo Borges, Luciano dos Santos Aggum Capettini, Adaliene Versiani Matos Ferreira, Daniella Bonaventura

**Affiliations:** ^1^Department of Pharmacology, Biological Sciences Institute, Federal University of Minas Gerais, Belo Horizonte, Brazil; ^2^Department of Biochemistry and Immunology, Biological Sciences Institute, Federal University of Minas Gerais, Belo Horizonte, Brazil; ^3^Department of Biological Sciences, Morphophysiology & Pathology Sector, Federal University of São Paulo, São Paulo, Brazil; ^4^Department of Nutrition, Nursing School, Federal University of Minas Gerais, Belo Horizonte, Brazil

**Keywords:** PVAT, obesity, high-carbohydrate diet, renin-angiotensin system, nitric oxide, hydrogen peroxide

## Abstract

The perivascular adipose tissue (PVAT) is an active endocrine organ responsible for release several substances that influence on vascular tone. Increasing evidence suggest that hyperactivation of the local renin-angiotensin system (RAS) in the PVAT plays a pivotal role in the pathogenesis of cardiometabolic diseases. However, the local RAS contribution to the PVAT control of vascular tone during obesity is still not clear. Since the consumption of a high-carbohydrate diet (HC diet) contributes to obesity inducing a rapid and sustained increase in adiposity, so that the functional activity of PVAT could be modulated, we aimed to evaluate the effect of HC diet on the PVAT control of vascular tone and verify the involvement of RAS in this effect. For that, male Balb/c mice were fed standard or HC diet for 4 weeks. Vascular reactivity, histology, fluorescence, and immunofluorescence analysis were performed in intact thoracic aorta in the presence or absence of PVAT. The results showed that HC diet caused an increase in visceral adiposity and also in the PVAT area. Phenylephrine-induced vasoconstriction was significantly reduced in the HC group only in the presence of PVAT. The anticontractile effect of PVAT induced by HC diet was lost when aortic rings were previously incubated with angiotensin-converting enzyme inhibitor, Mas, and AT_2_ receptors antagonists, PI3K, nNOS, and iNOS inhibitors, hydrogen peroxide (H_2_O_2_) decomposing enzyme or non-selective potassium channels blocker. Immunofluorescence assays showed that both Mas and AT_2_ receptors as well as nNOS and iNOS isoforms were markedly expressed in the PVAT of the HC group. Furthermore, the PVAT from HC group also exhibited higher nitric oxide (NO) and hydrogen peroxide bioavailability. Taken together, these findings suggest that the anticontractile effect of PVAT induced by HC diet involves the signaling cascade triggered by the renin-angiotensin system through the activation of Mas and AT_2_ receptors, PI3K, nNOS, and iNOS, leading to increased production of nitric oxide and hydrogen peroxide, and subsequently opening of potassium channels. The contribution of PVAT during HC diet-induced obesity could be a compensatory adaptive characteristic in order to preserve the vascular function.

## Introduction

According to the World Health Organization (WHO), obesity is defined as abnormal or excessive fat accumulation in adipose tissue, which is not only a large storage for lipids but also a dynamic endocrine organ that secretes several bioactive substances (World Health Organization [WHO], 2016). The worldwide prevalence of obesity nearly tripled between 1975 and 2016 in which more than 1.9 billion adults were overweight and of those over 650 million adults were obese (World Health Organization [WHO], 2016). As the population is becoming increasingly overweight and obese, the typical Western diet that contains large amounts of lipids and refined carbohydrates has been of greater concern.

Different dietary approaches in animal models have been shown to be crucial to elucidate the mechanistic effects of specific diets in the development of obesity. The detrimental effect of high-fat diets is already well documented in previous studies demonstrating that the long-term administration of 40–60% fat diets promotes metabolic changes, increased adiposity, and plasma levels of proinflammatory cytokines (Flanagan et al., 2008; Gregersen et al., 2012). Similarly, the consumption of high-refined carbohydrate diets (HC diet) also induces metabolic disorders (Ferreira et al., 2011; Oliveira et al., 2013), but it is taken into less consideration. The HC diet showed induce a rapid and sustained increase in adiposity, glucose intolerance, low insulin sensitivity, and atherogenic dyslipidemia, contributing to the development of obesity and related diseases (Porto et al., 2011; Oliveira et al., 2013).

Obesity is commonly related to a wide spectrum of cardiovascular diseases (Poirier et al., 2006; Koliaki et al., 2018). Although visceral adipose tissue is usually associated with a higher risk of cardiovascular diseases, there is a potential interest to study the role of fat accumulation around blood vessels in the pathogenesis of vascular dysfunction. The perivascular adipose tissue (PVAT) surrounds the adventitious layer of blood vessels in several vascular beds. It not only acts as a structural support and protection for most blood vessels, but it also secretes a variety of bioactive molecules that influence on vascular tone and on susceptibility to the pathogenesis of cardiovascular diseases related to obesity (Gao, 2007; Szasz et al., 2013; Majesky, 2015).

Due to its high plasticity through changes in adipocyte morphology or balance of vasoactive factors secreted, the PVAT is able to adapt to different physiological and pathological conditions (Galvez-Prieto et al., 2012; Van de Voorde et al., 2014). Under physiological conditions, the PVAT usually induces an anticontractile effect secreting predominantly vasodilator substances such as adiponectin (Fesus et al., 2007), leptin (Dashwood et al., 2011), angiotensin 1-7 (Lee et al., 2009), hydrogen peroxide (H_2_O_2_) (Gao et al., 2007), and nitric oxide (NO) (Malinowski et al., 2008). However, under pathological conditions, the PVAT can exhibit a vasoconstrictor profile secreting mainly angiotensin II (Galvez-Prieto et al., 2008) and superoxide anions (Ketonen et al., 2010). This plasticity of the PVAT is important for the maintenance of vascular homeostasis as it may influence on progression or regression of vascular diseases (Britton and Fox, 2011; Brown et al., 2014).

The mechanisms that mediate the role of PVAT on the control of vascular tone during obesity are still under study. Increasing evidence suggest that hyperactivation of the local renin-angiotensin system (RAS) in the PVAT plays a pivotal role in the pathogenesis of cardiometabolic diseases (Aghamohammadzadeh et al., 2012), since the essential components of RAS have been shown to be expressed in PVAT, specially AT_1_ and AT_2_ receptors (Galvez-Prieto et al., 2008), and also Mas receptors (Nobrega et al., 2019). AT_1_ receptors are responsible for most biological effects of angiotensin II, which includes vasoconstriction, sodium retention, aldosterone release, cell proliferation, cardiac and vascular hypertrophy, oxidative stress, and inflammation (Faria-Costa et al., 2014), whereas AT_2_ receptors have opposite effects that counterbalance those mediated by the classical activation of AT_1_ receptors (Rubio-Ruiz et al., 2014). However, the counter regulatory response to most of the deleterious effects of AT_1_ receptors is mainly attributed to angiotensin 1-7, which binds to Mas receptors (Bader et al., 2014) and also to AT_2_ receptors (Castro et al., 2005), promoting several protective effects in the vascular system, including vasodilation (Ren et al., 2002), reduction of oxidative stress (Raffai et al., 2011) and anti-inflammatory effects (Lee et al., 2015), especially in pathological conditions.

Given that the local RAS contribution to the role of PVAT on the vascular tone during obesity needs to be better elucidated, and that the relationship between obesity induced by HC diet and PVAT has not yet been investigated, we aimed to evaluate the effect of HC diet on the PVAT control of vascular tone and verify the involvement of RAS in this effect.

## Materials and Methods

### Experimental Animals and Dietary Treatment

All protocols with animal study were conducted in accordance with the Brazilian Council of Animal Research Guidelines (CONCEA), reviewed and approved by the Ethics Committee on Animal Use of Federal University of Minas Gerais (UFMG) under the protocol number 225/2013. Male Balb/c mice, 8 weeks of age, were obtained from the Center of Bioterism of Biological Sciences Institute of UFMG and kept under controlled conditions of temperature and luminosity (light-dark cycle of 12 h), with free access to water and food.

The animals were randomly divided into two groups: control and HC. The control group received a standard diet (Nuvilab CR-1), while the HC group received a refined carbohydrate enriched diet (HC diet) for 4 weeks. The HC diet was prepared using 45% (395 g) of the standard diet (powder), added to 45% (395 g) of condensed milk and 10% (83.79 g) of refined sugar, mixed until it forms a homogeneous mass to make small pellets. The macronutrient composition of the standard diet (4.0 kcal/g) was 65.8% carbohydrate, 3.1% fat, and 31.1% protein, obtained from the manufacturer’s information, while the macronutrient composition of the HC diet (4.4 kcal/g) was 74.2% carbohydrate, 5.8% fat, and 20% protein, obtained from the nutritional analysis carried out by Oliveira et al. (2013).

### Assessment of Body Weight, Food Intake, and Adiposity Index

Animals were weighed once a week and the food intake was measured twice a week. Samples of epididymal, retroperitoneal, and mesenteric adipose tissues were weighed to evaluate the adiposity index, according to the equation below (Oliveira et al., 2013).

$$AdiposityIndex\left(\%\right)=\frac{\sum A\,d\,i\,p\,o\,s\,e\,T\,i\,s\,s\,u\,e\,s\,W\,e\,i\,g\,h\,t}{A\,n\,i\,m\,a\,l\,W\,e\,i\,g\,h\,t}\times 100$$

### Vascular Reactivity

Animals were euthanized by decapitation and the thoracic aorta was carefully isolated and sectioned into two rings with 3 mm length each. In one of the rings the PVAT was completely removed while the other was kept intact. The aortic rings were placed between two stainless-steel stirrups and connected to an isometric tension transducer (World Precision Instruments, Inc., Sarasota, FL, United States). The vessels were placed in organ chambers containing modified Krebs–Henseleit physiological solution (mmol/L: NaCl 135.0; KCl 5.0; KH_2_PO_4_ 1.17; CaCl_2_ 2.5; MgSO_4_ 1.4; NaHCO_3_ 20.0; glucose 11.0) at 37°C with a stable pH 7.4 and gassed with carbogenic mixture (95% O_2_ and 5% CO_2_) (White Martins, Brazil). After 1 h of stabilization at a basal tension of 4.9 mN (0.5 g), the vessels were stimulated with potassium chloride (9 × 10^–2^ mol/L) in order to determine its viability. Subsequently, the aortas were previously contracted with phenylephrine (EC_50_ PE: 10^–7^ mol/L) and the presence of a functional endothelium was verified by the addition of acetylcholine (EC_50_ ACh: 10^–6^ mol/L). The endothelial integrity was considered in a minimum of 80% relaxation for acetylcholine. To assess the effect of HC diet on endothelium-dependent vasodilation, cumulative concentration-response curves for acetylcholine (ACh 10^–10^–10^–4^ mol/L) were obtained in aortas previously contracted with phenylephrine (EC_50_ PE: 10^–7^ mol/L) in the presence or absence of PVAT. Acetylcholine-induced vasodilation was expressed in percentage of relaxation. The effect of HC diet on vascular contractility was assessed in cumulative concentration-response curves for phenylephrine (PE 10^–10^–10^–4^ mol/L) obtained in endothelium-intact aortas in the presence or absence of PVAT. Phenylephrine-induced vasoconstriction was expressed in mN.

In order to investigate the mechanisms underlying the effects of HC diet on the PVAT control of vascular tone, cumulative concentration-response curves for phenylephrine (PE 10^–10^ –10^–4^ mol/L) were only performed in the presence of PVAT previously incubated for 30 min with one of the following drugs: captopril (10^–5^ mol/L - angiotensin converting enzyme inhibitor) (Kikta and Fregly, 1982; Su et al., 2008), A779 (10^–6^ mol/L – selective Mas receptor antagonist) (Peiró et al., 2013), PD123,319 (10^–6^ mol/L – selective AT_2_ receptor antagonist) (Su et al., 2008), LY294,002 (10^–6^ mol/L – PI3K inhibitor) (Jimenez et al., 2010), L-NAME (10^–4^ mol/L - non-selective NOS inhibitor) (Araújo et al., 2012), L-NNA (10^–6^ mol/L – selective eNOS inhibitor) (Araújo et al., 2012; Gonzaga et al., 2018; Nobrega et al., 2019), 1,400 W (10^–5^ mol/L - selective iNOS inhibitor) (Garvey et al., 1997), 7Ni (10^–4^ mol/L - selective nNOS inhibitor) (Babbedge et al., 1993), catalase (300 U/mL – catalyzes the decomposition of hydrogen peroxide) (Gonzaga et al., 2018) or tetraethylammonium (TEA, 10^–3^ mol/L – non-selective blocker of potassium channels) (Bonaventura et al., 2011). All concentrations of drugs were based on previous studies abovementioned. Agonist potencies and maximal responses were analyzed and expressed as pD_2_ (−log EC_50_) and Emax (maximum effect elicited by the agonist), respectively.

### Histological Analysis

Thoracic aortas were fixed in phosphate buffered formaldehyde solution for 48 h and then dehydrated in ascending concentrations (70, 80, and 90% and absolute I, II, and III) of ethyl alcohol, followed by diaphanization in xylol I, II, and III, and embedded in paraffin. 5 μm transversal sections were stained by hematoxylin-eosin for morphological analysis or picrosirius for quantification of collagen fibers. The area of the middle layer and the PVAT were quantified surrounding the region occupied by the middle layer in the thoracic aorta or the fractions of adipose tissue located around the adventitia, respectively, in a Leica microscope coupled to a Quantimet 500 image analysis system (Leica, Bannockburn, IL) using a × 5 lens magnification under a common light. Picrosirius-stained sections were examined in the same image analysis system aforementioned using a × 20 lens magnification. The area occupied by collagen was quantified by the color-detecting mode of the computer program in the adventitia. The aspect of collagen fibers was evaluated under a polarized light, allowing evaluation of the molecular disposition of collagen fibers (Borges et al., 2007; de Figueiredo Borges et al., 2008).

### Immunostaining of Mas and AT_2_ Receptors and the Isoforms of Nitric Oxide Synthase

Frozen thoracic aortas of control and HC groups were serially cut in 10 μm transversal sections, fixed in cold 100% acetone and washed with phosphate buffered saline (PBS). The fixed cryosections were rinsed in wash buffer (4% BSA + 0.1%Triton X-100, in PBS). Following appropriate blocking procedures (3% BSA in PBS), the slides were incubated overnight at 4°C with rabbit monoclonal anti-Mas (Alomone Labs Cat# AAR-013, RRID:AB_2039972), rabbit anti-AT_2_ (Alomone Labs Cat# AAR-012-AG, RRID:AB_2039724), mouse anti-eNOS (Santa Cruz Biotechnology Cat# sc-136977, RRID:AB_2267282), mouse anti-iNOS (Santa Cruz Biotechnology Cat# sc-7271, RRID:AB_627810), mouse anti-nNOS (Santa Cruz Biotechnology Cat# sc-5302, RRID:AB_626757), followed by incubation with goat anti-mouse secondary antibody conjugated with Alexa Fluor 488 (Santa Cruz Biotechnology Cat# sc-362257, RRID:AB_10989084) and goat anti-rabbit secondary antibody conjugated with Alexa Fluor 594 (Thermo Fisher Scientific Cat# A-11037, RRID:AB_2534095). The sections were examined under Nikon Eclipse Ti microscope (Nikon, United States) with excitation at 488/594 nm and emission at 520/600 nm. The fluorescence intensity emitted was measured in different fields with the same area and analysis parameters only in the PVAT of the control and HC groups using ImageJ^®^ software (NIH, Bethesda, MD, United States) and expressed as fold increase (Navia-Pelaez et al., 2017).

### Determination of Basal Nitric Oxide and Hydrogen Peroxide Availability

Fluorescent probes 4-amino-5-methylamino-2′,7′-difluorescein diacetate (DAF-2DA) and 2′,7′-dichlorodihydrofluorescein diacetate (DCF-DA) were used to measure NO and H_2_O_2_ *in situ*, respectively, in the PVAT of the control and HC groups. For that, thoracic aortas with intact PVAT of both experimental groups were embedded in freezing medium (Tissue-Tek, Sakura Finetek, Torrance, CA, United States). Transversal sections (10 μm thick) of frozen thoracic aortas were incubated with DAF-2DA (2.5 μmol/L) or DCF-DA (2.5 μmol/L) at 37°C, protected from light. The images were captured on a Zeiss Axio Imager A2 fluorescence microscope where DAF-2DA was excited at 488/519 nm, and DCF-DA was excited at a 590/618 nm. The fluorescence intensity emitted was measured in different fields with the same area and analysis parameters only in the PVAT of the control and HC groups using ImageJ^®^ software (NIH, Bethesda, MD, United States) and expressed as fold increase (Campos-Mota et al., 2017).

### Statistical Analysis

Graphs and analysis were blinded performed in the GraphPad Prism version 8 (GraphPad Software, San Diego, CA, United States). Determinations of EC_50_ and Emax were performed using the non-linear regression method of least squares (Meddings et al., 1989). The concentration values that produced half maximal contraction amplitude, which was determined after log transformation of the normalized concentration–response curves, were reported as negative logarithm (pD_2_). The Emax values were considered as the maximal amplitude response reached in the concentration-response curves. Results were presented as standard mean ± error (SEM). After checking adherence to the normal distribution, statistical significance was determined using Student’s *t*-test for two group’s comparison or two-way analysis of variance (ANOVA) for multiple group comparisons as appropriate, followed by Holm–Sidak’s *post hoc* test. A value of *p* < 0.05 was considered statistically significant.

### Materials

The drugs phenylephrine (PE), captopril, A779, PD123,319, LY-294,002, Nω-Nitro-L-arginine methyl ester hydrochloride (L-NAME), Nω-Nitro-L-arginine (L-NNA), 1,400 W, 7-Nitroindazole (7-Ni), catalase and tetraethylammonium (TEA) were purchased from Sigma-Aldrich (St. Louis, MO, United States). DAF-2DA and DCF-DA fluorescent probes were obtained from Invitrogen (Carlsbad, CA, United States).

## Results

### Food Consumption, Body Weight, and Adiposity Index

**FIGURE 1:**
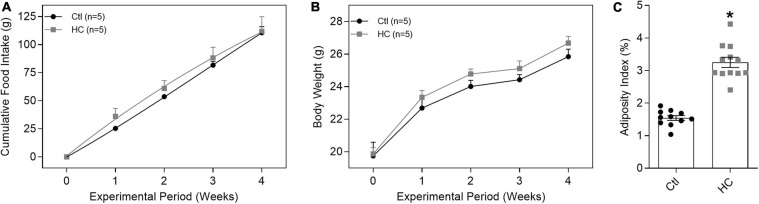
Evaluation of **(A)** cumulative food intake, **(B)** body weight, and **(C)** adiposity index of mice fed standard or HC diets. Values represent mean ± SEM. The total number of animals required to carry out the experiments in each group (*n*) is presented in parentheses or in the dispersion points in the graphs. Statistical significance was determined using **(A,B)** two-way ANOVA followed by Holm–Sidak’s *post hoc* test or **(C)** Student’s *t*-test. *p < 0.05 vs. control.

Despite no change in cumulative food intake (Figure 1A) and body weight (Figure 1B and Table 1), mice fed HC diet exhibited considerable increase in visceral adiposity (Figure 1C and Table 1).

**TABLE 1:** Body weight and epididymal (EAT), retroperitoneal (RAT), and mesenteric (MAT) adipose tissues values of control and HC groups.

Groups	Initial weight (g)	Final weight (g)	*n*	EAT (g)	RAT (g)	MAT (g)	*n*
Control	19.74 ± 0.84	25.84 ± 0.45	5	0.283 ± 0.012	0.052 ± 0.004	0.114 ± 0.010	11
HC	19.87 ± 0.38	26.67 ± 0.40	5	0.566 ± 0.031*	0.143 ± 0.009*	0.252 ± 0.018*	12

Values represent mean ± SEM. Statistical significance was determined using Student’s *t*-test. *p < 0.05 vs. control.

### Vascular Morphology and Collagen Evaluation

**FIGURE 2:**
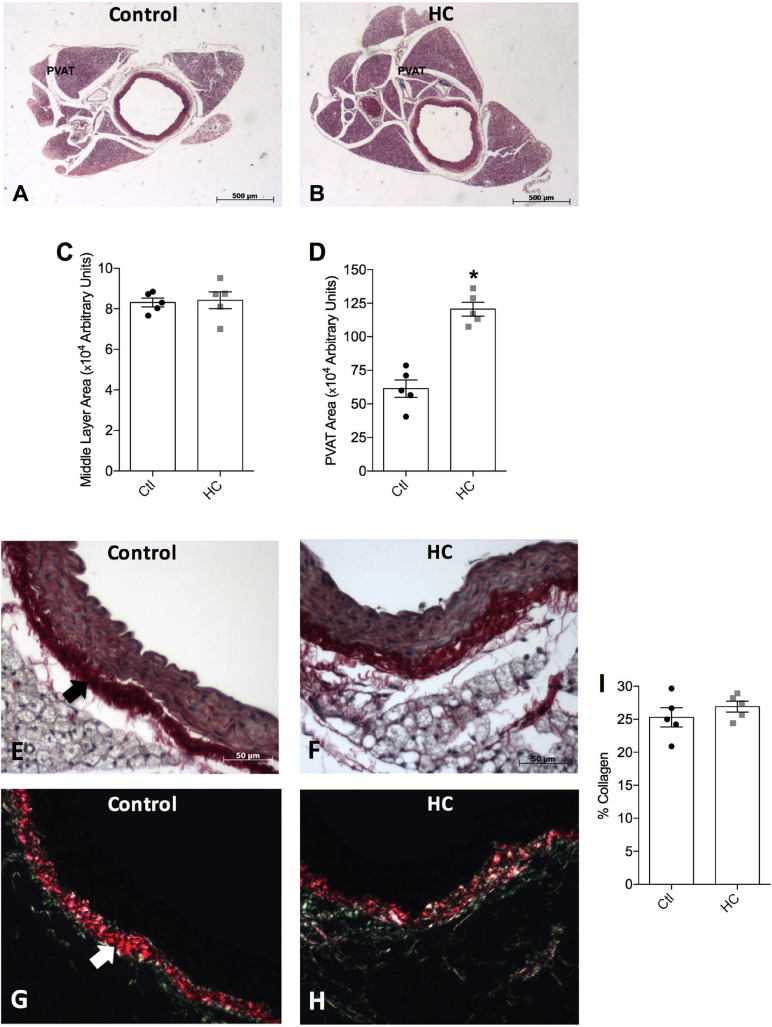
Histological analysis of thoracic aorta and PVAT of control and HC groups. Representative histological sections stained by **(A,B)** hematoxylin-eosin, or picrosirius under the incidence of **(E,F)** normal polychromatic light or **(G,H)** polarized light. The areas of **(C)** middle layer or **(D)** PVAT, and **(I)** the percentage of collagen fibers are represented in graphical bars with mean ± SEM. The total number of animals required to carry out the experiments in each group (*n*) is presented in the dispersion points in the graphs. Statistical significance was determined using Student’s *t*-test. *p < 0.05 vs. control. Arrows indicate the abundance of collagen fibers in the adventitious layer of blood vessels. Scale bars indicate **(A,B)** 500 μm or **(E–H)** 50 μm.

To verify whether HC diet induced an increase in the PVAT area, morphological analysis was performed. We verified in Figures 2A,B,D a significant increase in the area occupied by PVAT in the HC group when compared to the control group. No significant changes were verified in the middle layer area between the groups (Figures 2A–C).

Figure 2E shows the normal distribution of collagen fibers under the incidence of normal polychromatic light in the aorta of the control group. When evaluated under the incidence of polarized light, which allows analyzing the arrangement of the collagen fibers, Figure 2G shows that the collagen fibers were highly organized, exhibiting reddish color. Note the abundance of these fibers in adventitious layer (arrow). The same distribution and organization of the collagen fibers were observed in the aorta of the HC group (Figures 2F,H). Therefore, the HC diet did not induce vascular fibrosis, since no increase in the percentage of collagen fibers in the aorta of the HC group was found when compared to the control group (Figure 2I).

### Vascular Relaxation Induced by ACh

**FIGURE 3:**
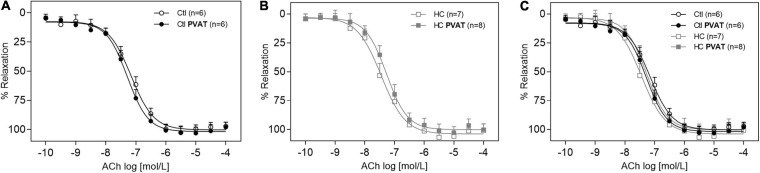
Vasodilator response induced by ACh in the presence or absence of PVAT in aortas of control and HC groups. Cumulative concentration-response curves for ACh in the presence or absence of PVAT in aortas of **(A)** control, **(B)** HC, and **(C)** overlapping curves of control and HC groups. Values represent mean ± SEM. The total number of animals required to carry out the experiments in each group (*n*) is presented in parentheses in the graphs. Statistical significance was determined using two-way ANOVA followed by Holm–Sidak’s *post hoc* test.

The endothelium-dependent vasodilation induced by ACh in the control group was similar in the presence or absence of PVAT (Figure 3A). The same result was found in the HC group (Figure 3B). The overlapping curves showed that the HC diet did not induce endothelial dysfunction when compared to the control group (Figure 3C). The Emax and pD_2_ values can be visualized in Table 2.

**TABLE 2:** Emax and pD_2_ values of vascular relaxation induced by ACh in intact thoracic aortas in the presence or absence of PVAT.

Groups	Emax (mN)	pD_2_ (−log EC_50_)	*n*
Control	97.11 ± 3.34	7.14 ± 0.09	6
HC	100.84 ± 5.50	7.54 ± 0.16	7
Control PVAT	97.98 ± 4.13	7.28 ± 0.07	6
HC PVAT	100.17 ± 5.04	7.32 ± 0.16	8

*Values represent mean ± SEM. Statistical significance was determined using two-way ANOVA followed by Holm–Sidak’s *post hoc* test.*

### Vascular Contraction Induced by PE

**FIGURE 4:**
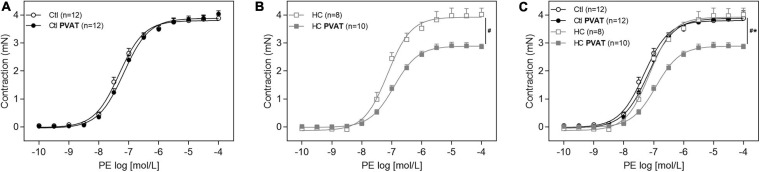
Contractile response induced by PE in the presence or absence of PVAT in aortas of control and HC groups. Cumulative concentration-response curves for PE in the presence or absence of PVAT in aortas of **(A)** control, **(B)** HC, and **(C)** overlapping curves of control and HC groups. Values represent mean ± SEM. The total number of animals required to carry out the experiments in each group (*n*) is presented in parentheses in the graphs. Statistical significance was determined using two-way ANOVA followed by Holm–Sidak’s *post hoc* test. *p < 0.05 vs. control PVAT; #*p* < 0.05 vs. HC PVAT represent the differences in the Emax values.

In Figure 4A, the presence of PVAT did not alter the vasoconstrictor response induced by PE in aortas of the control group. However, after HC diet for 4 weeks, the presence of PVAT significantly attenuated PE-induced vasoconstriction (Figure 4B). The overlapping curves showed that, in the absence of PVAT, the vasoconstriction induced by PE was similar between both groups. Only in the presence of PVAT the contractile response induced by PE was significantly reduced in the HC group (Figure 4C).

Once the HC diet attenuated the vascular contraction induced by PE only in the presence of PVAT, the next experiments were performed in aortas with intact PVAT in order to identify the mechanisms underlying the anticontractile effect induced by HC diet. All the Emax and pD_2_ values can be visualized in Table 3.

**TABLE 3:** Emax and pD_2_ values of vascular contraction induced by PE in intact thoracic aortas in the presence or absence of PVAT, previously incubated or not with the specified drugs.

Groups	Emax (mN)	pD_2_ (−log EC_50_)	*n*
Control	3.88 ± 0.10	7.18 ± 0.06	12
HC	4.02 ± 0.22^#^	7.18 ± 0.12	8
Control PVAT	4.04 ± 0.11	7.00 ± 0.07	12
HC PVAT	2.86 ± 0.09*	6.94 ± 0.06	10
Control PVAT Captopril	3.68 ± 0.22	7.32 ± 0.09	7
HC PVAT Captopril	3.67 ± 0.16^#^	7.20 ± 0.09	5
Control PVAT A779	3.85 ± 0.17	7.52 ± 0.05*	8
HC PVAT A779	4.07 ± 0.10^#^	7.44 ± 0.11^#^	8
Control PVAT PD123,319	4.13 ± 0.11	7.42 ± 0.04*	5
HC PVAT PD123,319	4.23 ± 0.14^#^	7.36 ± 0.15^#^	5
Control PVAT LY294,002	3.84 ± 0.31	7.20 ± 0.17	6
HC PVAT LY294,002	3.91 ± 0.11^#^	7.23 ± 0.08	10
Control PVAT L-NAME	4.04 ± 0.06	7.66 ± 0.07*	6
HC PVAT L-NAME	4.01 ± 0.13^#^	7.82 ± 0.16^#^	11
Control PVAT L-NNA	3.82 ± 0.10	7.41 ± 0.03*	6
HC PVAT L-NNA	3.10 ± 0.09*∧	7.13 ± 0.05^∧^	6
Control PVAT 1,400 W	4.04 ± 0.13	7.54 ± 0.13*	5
HC PVAT 1,400 W	3.97 ± 0.19^#^	6.97 ± 0.08^∧^	6
Control PVAT 7Ni	4.16 ± 0.23	7.40 ± 0.07*	7
HC PVAT 7Ni	4.19 ± 0.19^#^	7.26 ± 0.09^#^	8
Control PVAT Catalase	3.92 ± 0.09	6.95 ± 0.06	5
HC PVAT Catalase	3.86 ± 0.11^#^	7.02 ± 0.05	6
Control PVAT TEA	3.72 ± 0.13	7.79 ± 0.16*	12
HC PVAT TEA	3.68 ± 0.07^#^	7.14 ± 0.10^∧^	6

Values represent mean ± SEM. Statistical significance was determined using two-way ANOVA followed by Holm–Sidak’s *post hoc* test. *p < 0.05 vs. control PVAT; #*p* < 0.05 vs. HC PVAT; ∧*p* < 0.05 vs. control PVAT L-NNA, 1,400 W or TEA.

### Involvement of Renin-Angiotensin System

**FIGURE 5:**
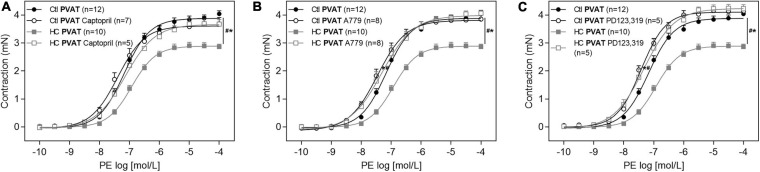
Involvement of RAS in the effect of HC diet on the PVAT control of vascular tone. Cumulative concentration-response curves for PE in the presence of PVAT in aortas of control and HC groups previously incubated or not with **(A)** captopril, **(B)** A779, or **(C)** PD123,319. Values represent mean ± SEM. The total number of animals required to carry out the experiments in each group (*n*) is presented in parentheses in the graphs. Statistical significance was determined using two-way ANOVA followed by Holm–Sidak’s *post hoc* test. *p < 0.05 vs. control PVAT; #*p* < 0.05 vs. HC PVAT represent the differences in the Emax and pD_2_ values.

As shown in Figure 5A, the anticontractile effect of PVAT induced by HC diet was lost when aortic rings were previously incubated with the angiotensin converting enzyme (ACE) inhibitor, captopril. In addition, we verified the involvement of RAS receptors in this anticontractile effect of PVAT. The antagonism of Mas (Figure 5B) and AT_2_ (Figure 5C) receptors with A779 and PD123,319, respectively, also reestablished the contractile response induced by PE in the HC group to the level found in the control group.

### Immunolocalization of Mas and AT_2_ Receptors

**FIGURE 6:**
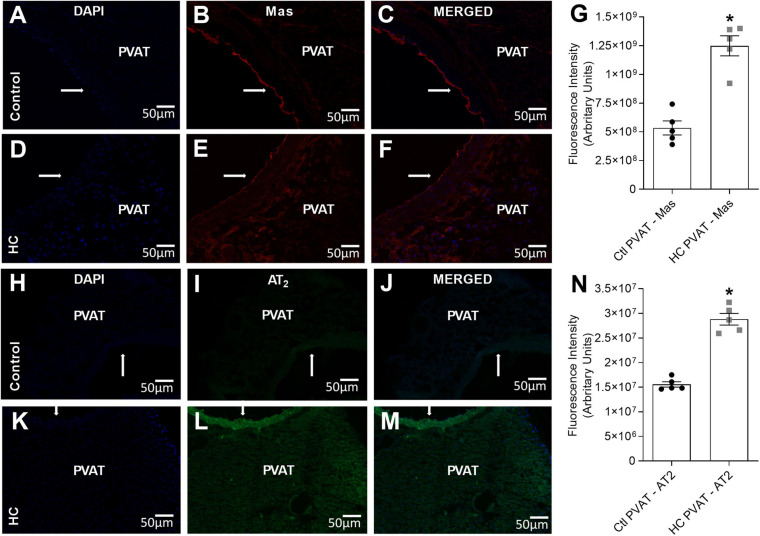
Immunofluorescence of Mas and AT_2_ receptors in the PVAT of control and HC groups. **(A,D,H,K)** Nuclear immunostaining with DAPI. Immunostaining for **(B,E)** Mas or **(I,L)** AT_2_ receptors. Overlap of immunostaining for **(C,F)** Mas receptors and DAPI or **(J,M)** AT_2_ receptors and DAPI. Fluorescence intensity emitted by binding the selective secondary antibody to **(G)** Mas (red fluorescence) or **(N)** AT_2_ (green fluorescence) receptors in the PVAT, respectively, were represented in graphical bars with mean ± SEM. The total number of animals required to carry out the experiments in each group (*n*) is presented in the dispersion points in the graphs. Statistical significance was determined using Student’s *t*-test. *p < 0.05 vs. control. Arrows indicate the location of the endothelial layer of blood vessels. Scale bars indicate 50 μm.

Since the activation of Mas and AT_2_ receptors was associated with the anticontractile effect of PVAT induced by HC diet, we further investigated if Mas and AT_2_ receptors were expressed in the PVAT of control and HC groups. Immunofluorescence assays allowed to evaluate whether PVAT express the antigen of Mas and AT_2_ receptors. The results demonstrated the presence of Mas (Figures 6B,E) and AT_2_ (Figures 6I,L) receptors in the PVAT of animals fed standard or HC diet. However, the fluorescence intensity was markedly higher in the PVAT of the HC group when compared to the control group as shown in Figures 6G,N.

### Involvement of the PI3k-Akt-NOS Pathway and Evaluation of Basal NO Availability

**FIGURE 7:**
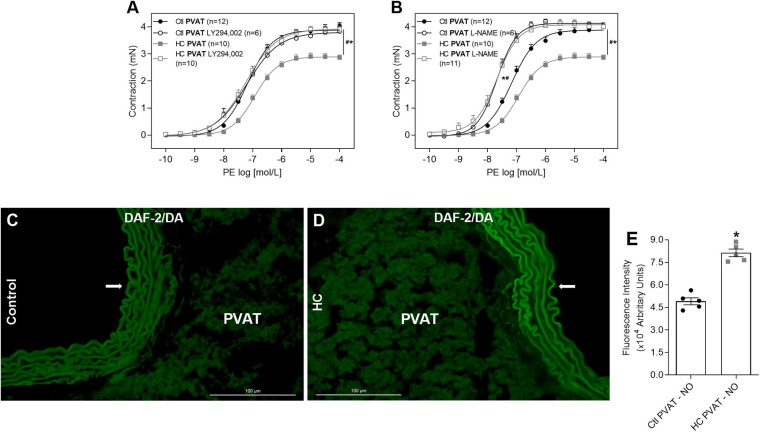
Involvement of PI3k-Akt-NOS pathway in the effect of HC diet on PVAT control of vascular tone and evaluation of NO availability. Cumulative concentration-response curves for PE in the presence of PVAT in aortas of control and HC groups previously incubated or not with **(A)** LY294,002 or **(B)** L-NAME. Values represent mean ± SEM. The total number of animals required to carry out the experiments in each group (*n*) is presented in parentheses. Statistical significance was determined using two-way ANOVA followed by Holm–Sidak’s *post hoc* test. *p < 0.05 vs. control PVAT; #*p* < 0.05 vs. HC PVAT represent the differences in the Emax and pD_2_ values. Representative sections of basal NO availability in the PVAT of **(C)** control and **(D)** HC groups exposed to DAF-2DA probe. Arrows indicate the location of the endothelial layer of blood vessels. Scale bars indicate 100 μm. **(E)** Quantification of NO production in the PVAT was expressed as fluorescence intensity in graphical bars with mean ± SEM. The total number of animals required to carry out the experiments in each group (*n*) is presented in the dispersion points in the graphs. Statistical significance was determined using Student’s *t*-test. *p < 0.05 vs. control.

As the activation of Mas and AT_2_ receptors can trigger the intracellular signaling cascade that activates PI3K-Akt pathway, we verified whether this pathway participates in the effect of HC diet on the control of vascular tone induced by PVAT. The inhibition of PI3K with LY294,002 reestablished the contractile response induced by PE in the HC group (Figure 7A). Knowing that the activation of PI3K-Akt pathway can lead to NOS phosphorylation, we evaluated the involvement of NOS in the anticontractile effect of PVAT induced by HC diet. As shown in Figure 7B, the non-selective inhibition of NOS with L-NAME reestablished the contractile response induced by PE in the HC group. Also, we verified in Figures 7C–E that basal NO availability was significantly higher in the PVAT from HC group when compared to the control group.

### Contribution of the Endothelial (eNOS), Inducible (iNOS), and Neuronal (nNOS) Isoforms of NOS

**FIGURE 8:**
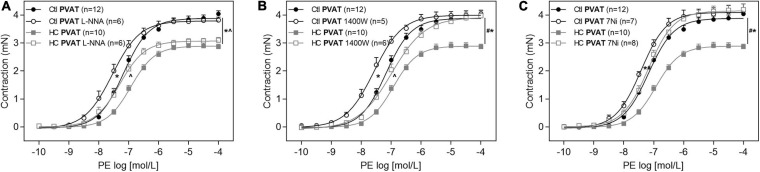
Implication of eNOS, iNOS, and nNOS in the effect of HC diet on control of vascular tone induced by PVAT. Cumulative concentration-response curves for PE in the presence of PVAT in aortas of control and HC groups previously incubated or not with **(A)** L-NNA, **(B)** 1,400 W, or **(C)** 7Ni. Values represent mean ± SEM. The total number of animals required to carry out the experiments in each group (*n*) is presented in parentheses in the graphs. Statistical significance was determined using two-way ANOVA followed by Holm–Sidak’s *post hoc* test. *p < 0.05 vs. control PVAT; #*p* < 0.05 vs. HC PVAT; **∧***p* < 0.05 vs. control PVAT L-NNA or 1,400 W represent the differences in the Emax and pD_2_ values.

Once the involvement of NOS was confirmed, as well as an increase in basal levels of NO, we further investigated which NOS isoforms would be related to the anticontractile effect of PVAT induced by HC diet. The inhibition of eNOS with L-NNA was not able to reverse the contractile response induced by PE in the HC group (Figure 8A). The iNOS inhibition with 1,400 W reestablished the Emax of contractile response induced by PE in the HC group, but the difference in pD_2_ values between both groups implies that the reversal of contractile response was only partial (Figure 8B and Table 3). However, the nNOS inhibition with 7Ni completely reestablished the contractile response induced by PE in the HC group (Figure 8C).

### Immunolocalization of eNOS, iNOS, and nNOS

**FIGURE 9:**
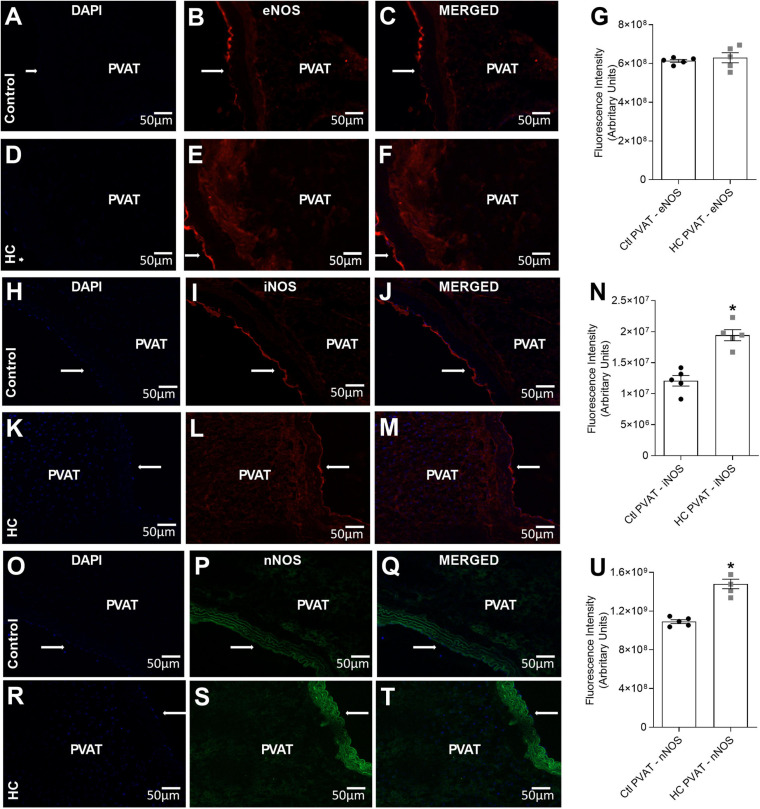
Immunofluorescence of eNOS, iNOS and nNOS in the PVAT of control and HC groups. **(A,D,H,K,O,R)** Nuclear immunostaining with DAPI. Immunostaining for **(B,E)** eNOS, **(I,L)** iNOS or **(P,S)** nNOS. Overlap of immunostaining for **(C,F)** eNOS and DAPI, **(J,M)** iNOS and DAPI or **(Q,T)** nNOS and DAPI. Fluorescence intensity emitted by binding the selective secondary antibody to **(G)** eNOS (red fluorescence), **(N)** iNOS (red fluorescence), or **(U)** nNOS (green fluorescence) in the PVAT, respectively, were represented in graphical bars with mean ± SEM. The total number of animals required to carry out the experiments in each group (*n*) is presented in the dispersion points in the graphs. Statistical significance was determined using Student’s *t*-test. *p < 0.05 vs. control. Arrows indicate the location of the endothelial layer of blood vessels. Scale bars indicate 50 μm.

To verify whether or not PVAT express the antigen of NOS isoforms in the PVAT of control and HC groups, immunofluorescence assays were performed and revealed the presence of eNOS (Figures 9B,E), iNOS (Figures 9I,L), and nNOS (Figures 9P,S) in the PVAT of animals fed standard or HC diet. As shown in Figure 9G, the fluorescence intensity for eNOS was similar between both groups. However, the fluorescence intensity for iNOS (Figure 9N) and nNOS (Figure 9U) were markedly higher in the PVAT of the HC group. These findings were in agreement with the results found in vascular reactivity experiments, since only iNOS and nNOS isorforms were involved in the anticontractile effect of PVAT induced by HC diet.

### Contribution of H_2_O_2_ and Potassium Channels

**FIGURE 10:**
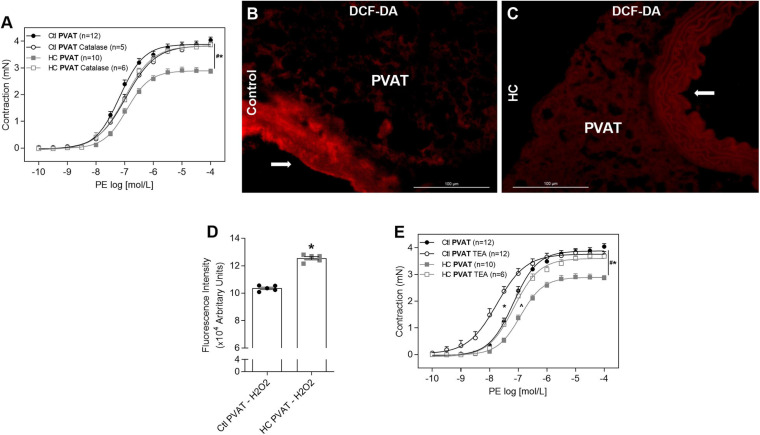
Involvement of H_2_O_2_ and potassium channels in the effect of HC diet on PVAT control of vascular tone and evaluation of H_2_O_2_ availability. Cumulative concentration-response curves for PE in the presence of PVAT in aortas of control and HC groups previously incubated or not with **(A)** catalase or **(E)** TEA. Values represent mean ± SEM. The total number of animals required to carry out the experiments in each group (*n*) is presented in parentheses in the graphs. Statistical significance was determined using two-way ANOVA followed by Holm–Sidak’s *post hoc* test. *p < 0.05 vs. control PVAT; #*p* < 0.05 vs. HC PVAT; **∧***p* < 0.05 vs. control PVAT TEA represent the differences in the Emax and pD_2_ values. Representative sections of basal H_2_O_2_ levels in the PVAT of **(B)** control and **(C)** HC groups exposed to DCF-DA probe. Arrows indicate the location of the endothelial layer of blood vessels. Scale bars indicate 100 μm. **(D)** Quantification of H_2_O_2_ production in the PVAT was expressed as fluorescence intensity in graphical bars with mean ± SEM. The total number of animals required to carry out the experiments in each group (*n*) is presented in the dispersion points in the graphs. Statistical significance was determined using Student’s *t*-test. *p < 0.05 vs. control.

Once we found that nNOS is the main isoform involved in the anticontractile effect of PVAT induced by HC diet, and as this isoform not only produces NO but also H_2_O_2_, we verified the involvement of H_2_O_2_, another potent vasodilator factor. The degradation of H_2_O_2_ with catalase reestablished the contractile response induced by PE in HC group (Figure 10A). As shown in Figures 10B–D, basal H_2_O_2_ availability was significantly higher in the PVAT from HC group when compared to the control group.

Also, to investigate if the vasodilator response induced by NO and H_2_O_2_ in the HC group involves hyperpolarization through the opening of potassium channels, aortic rings were previously incubated with TEA. The non-selective blockade of potassium channels with TEA reestablished the Emax of contractile response induced by PE in the HC group, but the difference in pD_2_ values between both groups implies that the reversal of contractile response was only partial (Figure 10E and Table 3).

## Discussion

Excessive consumption of high caloric density food, rich in lipids and refined carbohydrates, is largely responsible for the obesity epidemic associated with health complications including cardiovascular disease and metabolic syndrome (Azevedo and Brito, 2012; Ferreira et al., 2014). Indeed, our findings showed that mice fed a high-refined carbohydrate diet significantly increased visceral adiposity, despite the unchanged food intake and body weight. Similarly, Oliveira et al. (2013) showed that HC diet promotes rapid and sustained increase of visceral adiposity, perceptible from 1 day of diet and maintained for up to 12 weeks, even though the similarity in the food intake and body weight compared to mice that received a standard diet (Oliveira et al., 2013). Herein, we showed that HC diet also increased the PVAT area.

The adipose tissue is deeply related to the cardiovascular system. The understanding of this relationship has been widely advanced from studies involving the influence of the PVAT on the vascular function, focusing on the identification of bioactive molecules released under physiological and pathological conditions (Gu and Xu, 2013). In the present study, we sought to investigate the effect of HC diet on the control of vascular tone induced by PVAT.

Our results showed that the known anticontractile effect of PVAT was not observed in the vascular contraction induced by PE in the control group. Although several studies have demonstrated that the PVAT classically attenuates the contractile responses under physiological conditions in different vascular beds in both rodents and humans (Soltis and Cassis, 1991; Lohn et al., 2002; Dubrovska et al., 2004; Gao et al., 2005, 2007), this effect was not visualized in intact endothelium thoracic aorta of Balb/c mice which could be a limitation of the strain used in the present study, so comparisons to other studies must be done with care. Recently, Nobrega et al. (2019) found that the anticontractile effect of PVAT in thoracic aortas from Balb/c mice was only visualized in denuded endothelium aortas (Nobrega et al., 2019).

Interestingly, the HC diet significantly reduced the contractile response induced by PE only in the presence of PVAT, enhancing the anticontractile effect of PVAT once it was not observed in the control group as expected. These results corroborate those found in coronary arterioles of obese humans by Fulop et al. (2007), pioneers in suggesting that obesity may lead to the activation of adaptive vascular mechanisms to improve blood vessels function (Fulop et al., 2007). Moreover, our results showed that the HC diet did not induce endothelial dysfunction, since no impairment were found in endothelium-dependent vasodilation induced by ACh in the presence or absence of PVAT in the HC group when compared to the control group. However, our results differ from most studies that showed a vasoconstriction profile of PVAT and endothelial dysfunction during different diets-induced obesity, enriched in lipids or fructose, culminating in the loss of the anticontractile effect of PVAT (Ketonen et al., 2010; Ma et al., 2010; Rebolledo et al., 2010; Gil-Ortega et al., 2014). This might be due to the type of diet and the longer period of dietary treatment used in these studies.

Histological analysis showed that the attenuation of vascular contractility in the HC group only visualized in aortas with intact PVAT was not associated with a vascular fibrosis process. Therefore, we sought to investigate which factors could be responsible for enhancing the anticontractile effect of PVAT in the HC group.

The renin-angiotensin system (RAS) hyperactivity is one of the central mechanisms of cardiovascular diseases related to obesity (Rahmouni et al., 2005; Engeli, 2006). Several studies have been dedicated to investigate the local RAS, especially in the adipose tissue. Renin and all other components of the system (angiotensinogen, renin-binding protein, ACE, and peptidergic receptors), were found in adipose tissue of rodents and humans (Engeli et al., 1999; Schling et al., 1999; Cassis, 2000).

Herein, our results showed the involvement of ACE and the activation of Mas and AT_2_ receptors in the anticontractile effect of PVAT induced by HC diet. While the majority of studies address the effect of obesity associated with RAS hyperactivity through the ACE/Ang II/AT1 signaling pathway (Mathai et al., 2011), the ACE/Ang II/AT_2_ or ACE_2_/Ang 1-7/Mas/AT_2_ signaling pathways may be the key to elucidate the molecular mechanisms involved in protecting vascular homeostasis, especially during the development of pathological conditions such as obesity (Nguyen Dinh Cat and Touyz, 2011).

The literature has reported that activation of Mas and AT_2_ receptors can trigger the intracellular signaling cascade that activates the PI3K-Akt pathway (Sampaio et al., 2007; Wu et al., 2016). Also, in addition to the well-known activation of NOS by the calcium-calmodulin complex, alternative mechanisms of NOS activation have been proposed involving NOS phosphorylation through the PI3K-Akt signaling pathway (Fulton et al., 1999; Cunha et al., 2010). Therefore, we sought to investigate whether the components of this signaling pathway participated in the anticontractile effect of PVAT induced by HC diet. Our findings demonstrated that, besides the activation of Mas and AT_2_ receptors, the PI3K and NOS activation were also involved in the effect of HC diet on the control of vascular tone induced by PVAT, suggesting the activation of the signaling cascade triggered by RAS through the activation of Mas and AT_2_ receptors, PI3K-Akt and NOS in the anticontractile effect of PVAT induced by HC diet.

When we evaluated which NOS isoform was involved, our findings showed that the inhibition of eNOS did not reverse the effect of HC diet on the PVAT control of vascular tone. Only the inhibition of iNOS and nNOS isoforms partially and completely reestablished this effect, respectively. These results corroborated with the immunofluorescence assays that showed increased fluorescence intensity only of iNOS and nNOS isoforms in the PVAT of the HC group.

The iNOS isoform has been implicated in the pathogenesis of many diseases associated with inflammation, such as obesity (Fujimoto et al., 2005; Carvalho-Filho et al., 2009; Torrisi et al., 2016). In addition, recent studies have demonstrated that the PI3K/Akt pathway also leads to the activation of iNOS (Wang et al., 2015; Cianciulli et al., 2016). However, our results showed that the iNOS was partially involved in the anticontractile effect of PVAT induced by HC diet. The main isoform involved was the nNOS. Benkhoff et al. (2012) showed that aortic nNOS expression was increased in obese C57BL/6J mice fed a high-fat diet for 32 weeks (Benkhoff et al., 2012). Furthermore, the PI3K/Akt signaling pathway has also been shown to be involved in the activation of nNOS (El-Mas et al., 2009; Wu et al., 2016).

The nNOS not only produces NO but also H_2_O_2_, another potent vasodilator agent (Capettini et al., 2008). Our results showed the involvement of H_2_O_2_ in the anticontractile effect of PVAT induced by HC diet, with also increased basal levels of NO and H_2_O_2_ in the PVAT of the HC group. Both NO and H_2_O_2_ can induce vasodilator response in part through the opening of potassium channels (Barlow et al., 2000; Gao et al., 2003; Lee et al., 2009). We found that potassium channels were partially involved in the effect of HC diet on the control of vascular tone induced by PVAT. These findings suggest that, at least in part, NO and H_2_O_2_ could induce the anticontractile effect of PVAT in the HC group through the opening of potassium channels.

**FIGURE 11:**
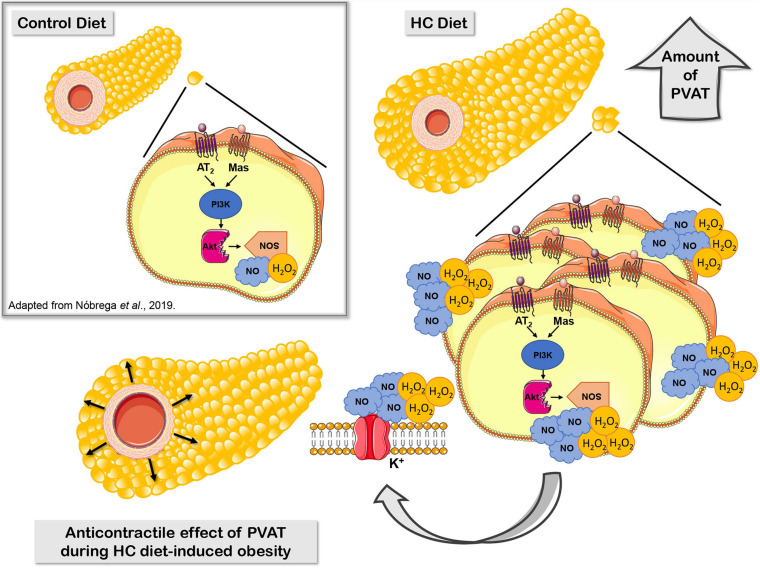
Proposed mechanisms underlying the anticontractile effect of PVAT during HC diet-induced obesity. Signaling cascade triggered by RAS through activation of Mas and AT_2_ receptors, PI3K, nNOS, or iNOS that lead to increased production of the vasodilator molecules NO and H_2_O_2_, and subsequently hyperpolarization by opening potassium channels.

Our research group recently demonstrated that, under physiological conditions, the activation of Mas and AT_2_ receptors and the production of H_2_O_2_ and NO contribute to the anticontractile effect of PVAT only visualized in denuded endothelium aortas (Nobrega et al., 2019). In the present study, we proposed that the HC diet induces a significant increase in the PVAT area, which may correlates with an increased Mas and AT_2_ receptors and production of NO and H_2_O_2_ that enhanced the anticontractile effect of PVAT previously not observed in intact endothelium aortas from animals fed a standard diet (Figure 11).

In summary, our findings improve the understanding about the early effect of PVAT on the control of vascular tone in an obesity context. The HC diet for 4 weeks enhanced the release of vasodilators factors from PVAT, suggesting that this could be a compensatory adaptive characteristic in order to preserve the vascular function during initial steps of obesity. The mechanisms underlying the anticontractile effect of PVAT induced by HC diet may involve the signaling cascade triggered by RAS through the activation of Mas and AT_2_ receptors, PI3K, nNOS, and iNOS that lead to increased production of NO and H_2_O_2_, and subsequently opening of potassium channels.

## Data Availability Statement

The original contributions presented in the study are included in the article/supplementary material, further inquiries can be directed to the corresponding author/s.

## Ethics Statement

The animal study was reviewed and approved by Ethics Committee on Animal Use of Federal University of Minas Gerais under the protocol number 225/2013.

## Author Contributions

DR: conceptualization, methodology, investigation, formal analysis, and writing – original draft. AS and GC: methodology and formal analysis. NN and NA: validation, formal analysis, and writing – review and editing. LF, LS, and AF: methodology, formal analysis, and resources. DB: conceptualization, supervision, resources, funding acquisition, and writing – review and editing. All authors contributed to the article and approved the submitted version.

## Conflict of Interest

The authors declare that the research was conducted in the absence of any commercial or financial relationships that could be construed as a potential conflict of interest.

## Abbreviations

ACh: acetylcholine
Ang II: angiotensin II
Ang 1-7: angiotensin 1-7
AT_2_: angiotensin II receptor type II
CaCl_2_: calcium chloride
Emax: maximum effect
eNOS: endothelial nitric oxide synthase
HC diet: high-carbohydrate diet
H_2_O_2_: hydrogen peroxide
iNOS: inducible nitric oxide synthase
KCl: potassium chloride
KH_2_PO_4_: monopotassium phosphate
Mas: angiotensin 1–7 receptor
MgSO_4_: magnesium sulphate
NaCl: sodium chloride
NaHCO_3_: sodium bicarbonate
nNOS: neuronal nitric oxide synthase
NO: nitric oxide
pD_2_: potency
PE: phenylephrine
PI3K: phosphatidylinositol 3-kinase
PVAT: perivascular adipose tissue
RAS: renin-angiotensin system
TEA: tetraethylammonium.
